# The functional epistasis of 5-HTTLPR and BDNF Val66Met on emotion processing: a preliminary study

**DOI:** 10.1002/brb3.99

**Published:** 2012-10-03

**Authors:** Tim Outhred, Pritha Das, Carol Dobson-Stone, Kristi Griffiths, Kim L Felmingham, Richard A Bryant, Gin Malhi, Andrew H Kemp

**Affiliations:** 1 School of Psychology, University of SydneyNew South Wales, 2006, Australia; 2 Discipline of Psychiatry, Sydney Medical School, University of Sydney, Royal North Shore HospitalNew South Wales, 2065, Australia; 3 CADE Clinic, Department of Psychiatry, Royal North Shore HospitalNew South Wales, 2065, Australia; 4 Advanced Research and Clinical Highfield Imaging (ARCHI), University of Sydney, Royal North Shore HospitalNew South Wales, 2065, Australia; 5 Neuroscience Research AustraliaRandwick, New South Wales, 2031, Australia; 6 School of Medical Sciences, University of New South WalesKensington, New South Wales, 2033, Australia; 7 School of Psychology, University of TasmaniaHobart, Tasmania, 7001, Australia; 8 School of Psychology, University of New South WalesKensington, New South Wales, 2033, Australia

**Keywords:** 5-HTTLPR, BDNF Val66Met, emotion processing, epistasis, fMRI, healthy subjects

## Abstract

An epistatic interaction of 5-HTTLPR and BDNF Val66Met polymorphisms has been implicated in the structure of rostral anterior cingulate cortex (rACC) and amygdala (AMY): key regions associated with emotion processing. However, a functional epistasis of 5-HTTLPR and BDNF Val66Met on overt emotion processing has yet to be determined. Twenty-eight healthy, Caucasian female participants provided saliva samples for genotyping and underwent functional magnetic resonance imaging (fMRI) during which an emotion processing protocol were presented. Confirming the validity of this protocol, we observed blood oxygen level–dependent (BOLD) activity consistent with fMRI meta-analyses on emotion processing. Region-of-interest analysis of the rACC and AMY revealed main effects of 5-HTTLPR and BDNF Val66Met, and an interaction of 5-HTTLPR and BDNF Val66Met. The effect of the BDNF Met66 allele was dependent on 5-HTTLPR alleles, such that participants with S and Met alleles had the greatest rACC and AMY activation during the presentation of emotional images relative to other genetic groupings. Increased activity in these regions was interpreted as increased reactivity to emotional stimuli, suggesting that those with S and Met alleles are more reactive to emotional stimuli relative to other groups. Although limited by a small sample, this study contributes novel and preliminary findings relating to a functional epistasis of the 5-HTTLPR and BDNF Val66Met genes in emotion processing and provides guidance on appropriate methods to determine genetic epistasis in fMRI.

## Introduction

The imaging genetics framework provides a methodological approach to examine the impact of genetic variation on the structure and function of brain regions involved in emotion processing (Hariri et al. 2006; Pezawas and Meyer-Lindenberg 2010). Many imaging genetics studies have now examined the roles of serotonin transporter (5-HTTLPR, e.g., Hariri et al. 2005; Hariri and Holmes 2006) and brain-derived neurotropic factor (BDNF Val66Met, e.g., Montag et al. 2008; Mukherjee et al. 2011) genetic polymorphisms – independent from each other – on the structure and function of regions involved in emotion processing. A recent meta-analysis observed that the effect size of 5-HTTLPR is smaller than previously reported (Murphy et al. 2012) and another highlighted the inconsistent effects of BDNF Val66Met (Verhagen et al. 2010). Elucidating an epistatic interaction of the two genes may help to better understand the role of these polymorphisms in emotion processing. While the impact of genetic epistasis on brain structure has been examined (Pezawas et al. 2008), studies remain to examine epistatic effects on brain function. A previous report (Wang et al. 2012) attempted to investigate a potential epistasis; however, analyses were not conducted to allow for an epistatic interaction to be determined. This study also had a variety of other methodological limitations (see Outhred and Kemp 2012 for commentary). Building on previous work, we report the results of a human in vivo functional magnetic resonance imaging (fMRI) study on overt emotion processing, exploring the impact of 5-HTTLPR and BDNF Val66Met polymorphisms and a potential epistatic interaction in a homogenous sample of healthy Caucasian subjects.

Gene–gene epistatic interactions may better explain the complex differential brain and behavior correlates of the 5-HTTLPR and BDNF Val66Met polymorphisms. The impact of 5-HTTLPR polymorphisms may vary depending on BDNF Val66Met variation, such that Met allele reduces sensitivity to 5-HT signaling (Murphy et al. 2003; Martinowich and Lu 2008). Equally, 5-HTTLPR may moderate the effect of BDNF Val66Met: the S allele reduces the capacity for BDNF expression (Murphy et al. 2003; Martinowich and Lu 2008). A structural neuroimaging study (Pezawas et al. 2008) reported that these two genetic polymorphisms interact in amygdala (AMY) and the rostral anterior cingulate cortex (rACC). These regions play a key role in emotion processing: the AMY responds to motivationally salient, exteroceptive sensory stimuli, while the rACC is associated with emotion regulation and response preparation (Lindquist et al. 2012).

Building on previous work (Wang et al. 2012), we examined the impact of 5-HTTLPR and BDNF Val66Met and their epistasis on blood oxygen level–dependent (BOLD) activity in the rACC and AMY during emotion processing in a sample of healthy, unmedicated, female Caucasian participants, thus circumventing the potential for the moderating effects of illness, treatment, sex, ethnicity, and associated factors. Consistent with Wang et al. (2012), we hypothesized that (1) the 5-HTTLPR and BDNF Val66Met polymorphisms would both impact on emotion processing and (2) these polymorphisms would interact in an epistatic manner during the processing of emotional stimuli in rACC and AMY.

## Method

### Participants

A sample of 28 healthy Caucasian females with complete fMRI, genotyping, and questionnaire data sets were recruited for this study in order to exclude effects of gender and impact of ethnicity and to reduce overall sample heterogeneity. Exclusion criteria included history of physical brain injury, neurological or psychiatric disorder, or any other serious medical condition. In addition, participants were excluded if they reported use of psychoactive medications or any psychotherapy within the past 6 weeks. All participants provided written informed consent in accordance with National Health and Medical Research Council guidelines.

### Genotyping

DNA was extracted from saliva samples and 5-HTTLPR and rs25531 (given the differential impact of the La and Lg genotypes), and BDNF Val66Met genotypes were determined according to protocols described previously (Joffe et al. 2009; Bryant et al. 2010; Quinn et al. 2012). Genotypes were scored independently by two researchers. The functional 5-HTTLPR genotypes were categorized as “S/S” (*n* = 6; 21%), “S/L” (*n* = 14; 50%), and “L/L” (*n* = 8; 29%), and were found to be in Hardy–Weinberg equilibrium, χ^2^ < 0.001, *P* = 0.98. The BDNF genotypes Val/Val (*n* = 16; 57%), Val/Met (*n* = 10; 36%), and Met/Met (*n* = 2; 7%) were also found to be in Hardy–Weinberg equilibrium, χ^2^ = 0.06, *P* = 0.80.

In accordance with previous literature (e.g., Pezawas et al. 2008; Bhang et al. 2011), the total of 28 participants were divided into four groups on the basis of their functional 5-HTTLPR genotype and their BDNF Val66Met genotype. Groups were as follows: S and Met group (5-HTTLPR S/S or S/L plus BDNF Val/Met or Met/Met) *n* = 4, 14%; S and Val/Val group (S/S or S/L plus Val/Val) *n* = 5, 18%; L/L and Met group (L/L plus Val/Met or Met/Met) *n* = 8, 29%; L/L and Val/Val group (L/L plus Val/Val) *n* = 11, 39%.

### Emotion processing task

Participants viewed 90 pictures selected from the International Affective Picture System (IAPS; Lang et al. 2008). With regard to normative ratings of valence and arousal for females, emotional images were grouped into three valence categories: negative, neutral, and positive, with all images high on arousal levels in order to maximize the impact on underlying emotional circuitry. As a result of the high arousal ratings, the neutral category was renamed as “interesting.” The inclusion of the interesting category allows for the arousal between valence categories to be controlled such that the emotional images vary across valence, but not arousal. No significant differences were found between any category for brightness and contrast. Stimuli were presented in nine blocks consisting of 10 images of the same valence category. The order of block presentation was pseudo-randomized to avoid consecutive presentation of blocks with similar valence. Each image was presented for 6 sec, followed by a 3-sec fixation cross and a 3-sec nonemotional landscape image. Images were back-projected onto a screen via an LCD video projector and were viewed by subjects through a mirror fixed to the scanner's head coil. Participants were instructed to simply pay attention to the images on the screen and to avoid regulating their immediate response to emotional content.

### fMRI acquisition parameters

Imaging was performed using a 3.0 T Siemens Trio scanner. Thirty-six consecutive axial slices (4-mm thickness) parallel to the anterior–posterior commissure covering the whole brain were imaged using a T2*-weighted gradient echo EPI sequence (echo time [TE] = 32 msec; repetition time [TR] = 3000 msec; matrix = 64 × 64; flip angle = 90°). The field of view was 240 mm and the effective in plane functional spatial resolution was 3.75 mm. For each functional run, 360 volumes were collected after discarding the first six. For anatomical reference, high-resolution whole-brain images were also acquired: TR = 1570 msec; TE = 3.22 msec; flip angle = 15°; matrix 512 × 512 × 192 mm. Movement was minimized with padding, and an fMRI compatible eye movement system was used to ensure that participants attended to the stimuli and did not close their eyes during the experiment.

### Participant characteristics and IAPS ratings

We tested for group differences in age, menstrual phase, hormonal birth control use, handedness, education, and depression (PHQ-9; Kroenke et al. 2001) and anxiety (GAD-7; Spitzer et al. 2006) symptoms. This was performed to ensure sample homogeneity between 5-HTTLPR × BDNF Val66Met groupings and to avoid potential confounding factors. Due to logistical constraints, estradiol and progesterone levels could not be measured. In order to prevent interference of online ratings during emotion processing (see Phan et al. 2003), participants were asked to rate each image on nine-point Likert valence and arousal scales immediately after the scanning session.

### fMRI data analysis

#### Preprocessing

The imaging data was preprocessed and analyzed using the image processing routines implemented within the statistical parametric mapping software package, SPM8 (http://www.fil.ion.ucl.ac.uk/spm/software/spm8/; Wellcome Trust Centre for Neuroimaging). Images for each subject were first corrected for susceptibility-by-movement artifacts and then realigned to the first volume of the time series. Realigned images were spatially normalized into a standard stereotactic space (Montreal Neurologic Institute template) and smoothed with a Gaussian kernel (FWHM 8 mm) in order to minimize anatomical differences. The BOLD response at each voxel was modeled with a canonical hemodynamic response function and its temporal derivative.

#### Effects of emotional stimuli

For each participant, brain activation was examined for the contrasts of the emotional (positive, negative, and interesting) images relative to the nonemotional landscape images: emotional > nonemotional. These individual contrast images were then used in the second-level random effects model in order to determine regional responses for the whole sample. We conducted a whole-brain analysis in order to ensure the emotion processing task activated regions associated with emotional processing, and critically, our regions of interest (ROIs) including the bilateral rACC (operationally defined as the portion of anterior cingulate that lies anterior and superior to the genu of the corpus callosum, with the posterior boundary of *y* = +30 mm; Bryant et al. 2005) and the left and right AMY (as previously defined; Tzourio-Mazoyer et al. 2002; Maldjian et al. 2003). The rACC was defined bilaterally as clusters on the left or right rACC may be indistinguishable due to low spatial resolution at 3 mm. For all analyses, we employed an alpha level of *P* < 0.05 (partial volume, FDR-corrected) and a spatial extent of five or more voxels per cluster in order to control for type I error rates associated with multiple comparisons within the ROIs. The bilateral rACC and left and right AMY ROIs were subsequently employed in the analyses examining gene effects.

#### Total gene effects, main effect of 5-HTTLPR, and main effect of BDNF Val66Met effect on emotional stimuli

In order to determine whether there were effects of genotype, an omnibus analysis of variance (ANOVA) on the emotional > nonemotional contrast was performed for the ROIs rACC and AMY. Consequently, a second ANOVA was performed for the emotional > nonemotional contrast in order to determine whether there was an effect of the 5-HTTLPR genotype (S and L/L groups) within the rACC and AMY ROIs. These analyses were then followed up with independent samples *t*-tests in order to determine the directions of the effects. Similarly, a third ANOVA was performed in order to determine whether there was an effect of the BDNF Val66Met genotype (Met and Val/Val groups) within the rACC and AMY ROIs for the emotional > nonemotional contrast. In order to determine the direction of the effect, this ANOVA was then followed up with independent samples *t*-tests.

#### Interaction effect of 5-HTTLPR × BDNF Val66Met on emotional stimuli

In order to test our a priori hypotheses for the 5-HTTLPR × BDNF Val66Met epistasis, we employed a 2 (S and L/L groups) × 2 (Met and Val/Val groups) ANOVA. The 2 × 2 ANOVA was then followed up with independent samples *t*-tests in order to determine the directions of the effects across the four cells. We then extracted beta weights from each participant from their whole-brain emotional > nonemotional contrasts in order to inspect the distribution of these beta weights within and between each genotype cell. This data analysis strategy was performed in order to increase our confidence in the findings obtained from the small sample of genetic groupings relating to the interaction effects or genetic epistasis.

#### IAPS ratings and BOLD activation

In order to examine the relationship between subjective ratings of emotion processing and BOLD activation during emotion processing, a multiple regression was performed in IBM SPSS Statistics version 19. The dependent variable was the individual beta weights extracted from an exemplar ROI – the rACC was selected as its pattern of results was similar to that of the AMY – for the emotional > nonemotional contrasts. The predictors were the IAPS ratings of the valence and arousal ratings from the positive, negative, and interesting images and were entered altogether into the regression.

## Results

### Participant characteristics and IAPS ratings

Participant characteristics and IAPS ratings across 5-HTTLPR × BDNF Val66Met genotype groups are displayed in Table 1. There were no differences between groups in age, stage of menstrual phase, hormonal birth control use, handedness, education, or depression (PHQ-9) and anxiety (GAD-7) symptoms. Participants' ratings of the valence of the stimuli were congruent with the categories of positive, negative, and interesting. Additionally, ratings confirm that participants found the stimuli to be arousing consistent with normative ratings of the stimuli.

**Table 1:** Participant demographics and IAPS ratings for 5-HTTLPR × BDNF Val66Met allele grouping

	5-HTTLPR × BDNF Val66Met				
		
	S		L/L		
			
	Met (*n* = 4)	Val/Val (*n* = 5)	Met (*n* = 8)	Val/Val (*n* = 11)	Test of group differences
Age (years; SD)	22.00 (4.08)	25.60 (6.95)	27.25 (9.62)	23.09 (4.30)	*F*(3, 24) = 0.84; *P* = 0.48
Menstrual phase (M, N)	0/4	1/4	3/5	0/11	χ^2^ (3) = 6.15; *P* = 0.10
Hormonal birth control (N/Y)	4/0	2/3	5/3	7/4	χ^2^ (3) = 3.52; *P* = 0.32
Handedness (L/R)	0/4	1/4	0/8	2/9	χ^2^ (3) = 2.53; *P* = 0.47
Education (years; SD)	14.00 (2.65)	15.20 (2.49)	13.88 (1.73)	15.18 (2.86)	*F*(3, 23) = 0.58; *P* = 0.63
PHQ-9 (SD)	8.00 (7.00)	5.00 (6.86)	8.13 (8.68)	4.70 (5.12)	*F*(3, 22) = 0.48; *P* = 0.77
GAD-7 (SD)	7.33 (7.07)	5.40 (5.98)	5.75 (3.77)	4.90 (4.33)	*F*(3, 22) = 0.23; *P* = 0.88
Neg valence (SD)	2.58 (0.28)	2.37 (0.19)	2.65 (0.60)	2.56 (0.53)	*F*(3, 24) = 0.36; *P* = 0.79
Neg arousal (SD)	6.47 (0.84)	6.32 (1.40)	4.99 (2.04)	5.66 (1.33)	*F*(3, 24) = 1.179; *P* = 0.34
Int valence (SD)	4.74 (0.21)	4.45 (0.14)	4.71 (0.98)	4.44 (0.40)	*F*(3, 24) = 0.36; *P* = 0.79
Int arousal (SD)	5.31 (0.35)	5.22 (1.23)	4.38 (2.04)	4.42 (1.33)	*F*(3, 24) = 1.179; *P* = 0.34
Pos valence (SD)	5.98 (0.91)	6.85 (0.76)	6.40 (0.62)	6.73 (1.13)	*F*(3, 24) = 0.92; *P* = 0.45
Pos arousal (SD)	5.29 (0.47)	5.95 (1.27)	4.90 (1.46)	5.35 (1.20)	*F*(3, 24) = 0.75; *P* = 0.53

SD, standard deviation; M, mid-luteal; N, not mid-luteal; N, no birth control; Y, birth control; Neg, negative; Int, interesting; Pos, positive.

### fMRI results

#### Effects of emotional stimuli

Emotional stimuli activated regions including the inferior frontal gyrus, middle temporal gyrus, cuneus, precuneus, superior temporal gryus, middle occipital gyrus, middle frontal gyrus, superior parietal lobule, insula, cingulate cortex, caudate, cerebellum, thalamus, and AMY relative to the nonemotional landscape stimuli in the total sample of 28 participants, consistent with previous meta-analyses (Phan et al. 2002; Wager et al. 2010; Lindquist et al. 2012).

#### Total gene effects on emotional stimuli

In order to determine whether there are effects of genotype within the rACC and AMY, an ROI analysis on all genotype effects for the total sample (*N* = 28) was performed. The analysis revealed a significant bilateral rACC cluster (*k* = 102; peak voxel at [−12, 36, 24], *F* = 4.02, *P* < 0.001 [partial volume, FDR-corrected], η^2^ = 0.56), left AMY cluster activation (*k* = 47; peak voxel at [−27, −3, −18], *F* = 3.30, *P* = 0.003 [partial volume, FDR-corrected], η^2^ = 0.51), and right AMY cluster activation (*k* = 30; peak voxel at [21, −3, −18], *F* = 2.79, *P* = 0.026 [partial volume, FDR-corrected], η^2^ = 0.47).

#### Main effect of 5-HTTLPR on emotional stimuli

The rACC and AMY ROI analysis on the main effect of 5-HTTLPR (S, *n* = 9; L/L, *n* = 19) showed a significant bilateral rACC cluster (*k* = 370; peak voxel at [15, 39, 6], *F* = 12.57, *P* = 0.001 [partial volume, FDR-corrected], η^2^ = 0.27) and a left AMY cluster activation (*k* = 21; peak voxel at [−21, 0, −18], *F* = 8.32, *P* = 0.021 [partial volume, FDR-corrected], η^2^ = 0.20). Relative to L/L homozygotes, S carriers showed greater activation in the rACC (*k* = 231; peak voxel at [−12, 36, 24], *t* = 4.68, *P* < 0.001 [partial volume, FDR-corrected], *d* = 0.94) and a left AMY cluster activation (*k* = 42; peak voxel at [−27, −3, −15], *t* = 4.02, *P* < 0.001 [partial volume, FDR-corrected], *d* = 0.80). There were no significant activations for L/L homozygotes relative to S carriers.

#### Main effect of BDNF Val66Met on emotional stimuli

The rACC and AMY ROI analysis on the main effect of BDNF Val66Met (Met, *n* = 12; Val/Val, *n* = 16) showed a significant right AMY cluster activation (*k* = 21; peak voxel at [27, −3, −15], *F* = 14.63, *P* < 0.001 [partial volume, FDR-corrected], η^2^ = 0.31) and an rACC cluster activation (*k* = 31; peak voxel at [−9, 36, 15], *F* = 5.93, *P* = 0.019 [partial volume, FDR-corrected], η^2^ = 0.15). Relative to Val/Val homozygotes, Met carriers showed significantly greater activation in the right AMY cluster (*k* = 21; peak voxel at [27, −3, −15], *t* = 3.83, *P* < 0.001 [partial volume, FDR-corrected], *d* = 0.77) and an rACC cluster activation (*k* = 109; peak voxel at [−9, 36, 15], *t* = 2.43, *P* = 0.009 [partial volume, FDR-corrected], *d* = 0.49). Conversely, Val/Val showed no significant activations relative to Met carriers.

#### Interaction effect of 5-HTTLPR × BDNF Val66Met on emotional stimuli

**Figure 1:**
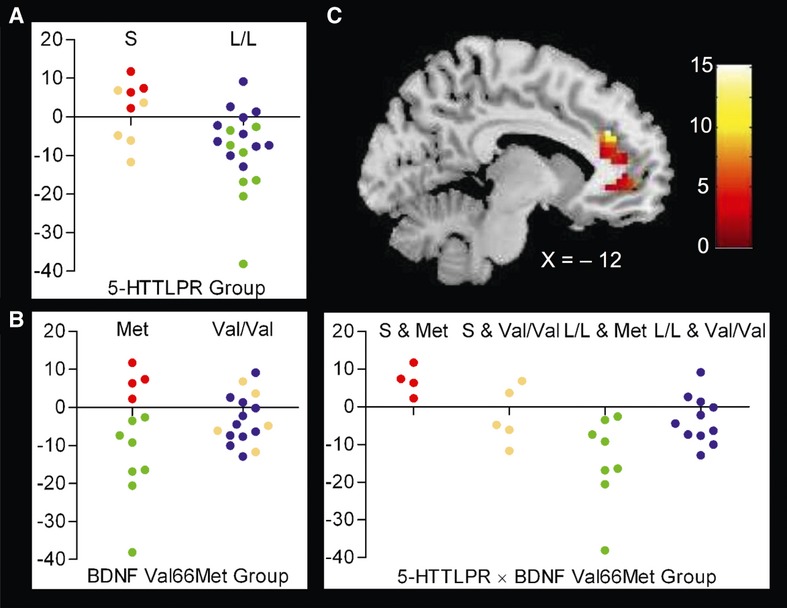
rACC activation to emotional stimuli relative to nonemotional stimuli. (A) Main effect of 5-HTTLPR. (B) Main effect of BDNF Val66Met. (C) Interaction effect of 5-HTTLPR × BDNF Val66Met, demonstrating epistasis. Red dots, S and Met group; Yellow dots, S and Val group; Green dots, L/L and Met group; Blue dots, L/L and Val/Val group.

The rACC and AMY ROI analysis on the 5-HTTLPR × BDNF Val66Met (S and Met, *n* = 4; S and Val/Val, *n* = 5; L/L and Met, *n* = 11; L/L and Val/Val, *n* = 8) interaction effect, with follow-up comparisons, is displayed in Table 2. Relative to all other groups, the S and Met group had greater activation in the rACC and AMY. Inspection of the distribution of beta weights between 5-HTTLPR × BDNF Val66Met cells demonstrated a clear interaction (as displayed in Fig. 1 with the rACC activation displayed as an exemplar as a similar distribution was found for the AMY). The activity of all the S and Met participants was increased and activity for all the L/L and Met participants was decreased, and the activity of S and Val/Val and L/L and Val/Val participants lay in between that of the former two genetic groupings.

**Table 2:** Peak rACC and AMY activation to emotional stimuli for 5-HTTLPR × BDNF Val66Met groupings

						Peak voxel (MNI)		
						
Group contrast	Region	*k*	Peak *t* value	*P*	Effect size *d*	*x*	*y*	*z*
Interaction effect	rACC	327	*F* =14.35	<0.001*	*r*^2^ = 0.54	−12	36	24
			*F* =7.10	<0.001†	*r*^2^ = 0.37	0	36	30
			*F* =6.82	<0.001†	*r*^2^ = 0.36	−9	51	0
	Left AMY	35	*F* =7.53	0.035*	*r*^2^ = 0.39	−27	0	−18
	Right AMY	28	*F* =6.68	<0.001†	*r*^2^ = 0.36	27	−3	−15
			*F* =4.63	0.003†	*r*^2^ = 0.28	33	0	−27
S and Met > L/L and Met	rACC	173	6.70	<0.001*	1.34	−9	36	27
			3.35	0.001†	0.67	−3	30	−3
			2.74	0.004†	0.55	−12	42	15
	Left AMY	45	4.94	0.027*	0.99	−30	−3	−18
	Right AMY	37	2.96	0.002†	0.59	27	−9	−12
			2.59	0.006†	0.52	30	3	−18
			2.45	0.009†	0.49	21	−3	−18
S and Val/Val > L/L and Met	Right AMY	40	4.89	0.029*	0.98	27	−6	−15
			2.16	0.018†	0.43	24	6	−18
	rACC	254	3.81	<0.001†	0.76	−9	39	24
			3.16	0.001†	0.63	−15	42	12
			3.10	0.002†	0.62	−9	51	0
	Left AMY	25	3.03	0.002†	0.61	−27	−3	−15
L/L and Val/Val > L/L and Met	rACC	278	4.57	0.045*	0.91	−9	36	27
			3.03	0.002†	0.61	3	48	27
			2.88	0.003†	0.58	0	30	−3
	Right AMY	33	3.77	0.001†	0.75	27	−3	−15
			2.63	<0.001†	0.53	18	−3	−15
	Left AMY	26	3.35	0.001†	0.67	−27	−3	−15
			2.42	0.010†	0.48	−18	−6	−18
S and Met > S and Val/Val	rACC	9	3.02	0.002†	0.60	−12	33	27
	Left AMY	14	2.44	0.009†	0.49	−30	−3	−18
S and Met > L/L and Val/Val	rACC	10	3.62	<0.001†	0.72	−12	33	27
	Left AMY	19	2.53	0.007†	0.51	−30	−3	−18
			1.96	0.028†	0.39	−21	0	−12
S and Val/Val > L/L and Val/Val	Right AMY	5	2.25	0.014†	0.45	27	−6	−15
S and Val/Val > S and Met	Right AMY	6	1.99	0.026†	0.40	27	−6	−15
L/L and Met > S and Met			n.s.					
L/L and Met > S and Val/Val			n.s.					
L/L and Met > L/L and Val/Val			n.s.					
L and Val > S and Met			n.s.					
L and Val > S and Val/Val			n.s.					

rACC, bilateral rostral anterior cingulate cortex; AMY, amygdala; *k*, cluster extent; n.s., no significant voxels; *d*, Cohen's *d*.

* Partial volume, FDR-corrected *P* value.

† Uncorrected *P* value.

#### IAPS ratings and BOLD activation

In order to examine the relationship between subjective ratings of emotion processing and BOLD activation during emotion processing, a multiple regression of the IAPS ratings and the distribution of rACC BOLD activation was conducted. The IAPS ratings significantly predicted 43% of the variance (adjusted *R*^2^ = 0.268) in BOLD activation of the rACC, *F*(6, 21) = 2.645, *P* = 0.045. Ratings of both valence (interesting) and arousal (negative) were significant predictors.

## Discussion

The aim of this study was to determine the functional effects of 5-HTTLPR, BDNF Val66Met, and whether their epistasis impacts on emotion processing. Building on previous research (Wang et al. 2012), the 5-HTTLPR and BDNF Val66Met polymorphisms were found to interact in the rACC and the AMY during overt emotion processing in a homogenous, healthy sample of Caucasian females. The effect of the BDNF Met66 allele was moderated by the 5-HTTLPR alleles such that S and Met carriers displayed the greatest activation of the rACC and AMY in response to emotional images, while L/L and Met carriers had the least. Therefore, the epistasis of 5-HTTLPR and BDNF Val66Met is not only related to structural variation of the rACC, as reported previously (Pezawas et al. 2008), it is also associated with functional variation. Relative to all other groups, participants with S and Met alleles are more reactive to emotional stimuli generally. Findings such as these may have implications for the understanding of affective disorder development and maintenance (Martinowich and Lu 2008; Grabe et al. 2012).

A particularly novel finding obtained in the present study is the observation of a potential epistatic relationship of the 5-HTTLPR and BDNF Val66Met polymorphisms during emotion processing. Our data indicate that the vulnerable effects of the Met66 allele are – at least – partially dependent on 5-HTTLPR polymorphisms, such that the S allele in combination with the Met66 allele is associated with the greatest activation, while the L allele in combination with the Met66 allele is associated with reduced activity (Fig. 1). Therefore, we suggest that the S and Met combination is the most vulnerable against all other combinations, while the L/L and Met may be the least vulnerable. Serotonergic activity is partly due to the modulatory effects on the serotonin transporter (Mössner et al. 2000). Low 5-HTT efficiency in S carriers may reduce BDNF Val66Met gene expression and the less efficient Met66 allele may magnify this effect (Mamounas et al. 2000; Murphy et al. 2003; Martinowich and Lu 2008). This effect may result from impaired neurogenesis of serotonergic neurons (Ren-Patterson et al. 2005) supporting the circuitry underlying emotional experiences (Mukherjee et al. 2011). In contrast, our data suggest that the more efficient L/L genotype may compensate for the effects of the Met66 allele. Our findings further highlight the need for future neurocellular research to consider the impact of 5-HTTLPR and BDNF Val66Met epistasis on the neurogenesis of emotion circuitry.

Our findings indicated that participants with a combination of 5-HTTLPR S and BDNF Met66 alleles display the greatest activity in rACC and AMY in response to high-arousal emotional images relative to nonemotional landscape images. We also found that participant ratings of emotional stimuli were strongly associated with rACC activation during the presentation of the stimuli. This finding further supports the notion that differential rACC activity may be associated with individual differences in adaptive emotion regulation and response preparation (e.g., Roiser et al. 2012). Prior studies have reported that harm avoidance and neuroticism – well-validated, heritable personality measures linked to the risk of affective disorders – are also associated with the S allele of 5-HTTLPR gene (Lesch et al. 1996; Reif and Lesch 2003; Sen and Burmeister 2004), the Met66 allele of the BDNF Val66Met gene (Gatt et al. 2009), and heightened rACC and AMY activity in response to emotional stimuli (Keightley et al. 2003; Bertolino et al. 2005; Pezawas et al. 2005). The epistasis of 5-HTTLPR and BDNF Val66Met influences susceptibility for dysfunctional affective disorder-related personality characteristics (Lang et al. 2005; Ren-Patterson et al. 2005). For instance, the number of risk alleles increases susceptibility for rumination, with those of the S/S and Met/Met genotype at the most risk (Clasen et al. 2011). Our results suggest that individual differences in emotional reactivity may be underpinned, in part, by the epistasis of BDNF Val66Met and 5-HTTLPR polymorphism variants. Future examination of the 5-HTTLPR and BDNF Val66Met epistasis on emotion processing also should consider associated risk factors such as personality traits. This line of enquiry may provide further insights into the development and maintenance of affective disorders.

Our finding that the S and Met group was the most reactive to emotional stimuli – suggesting that it is the most vulnerable group – is consistent with that of Wang and colleagues (2012). Although they did not test for an epistatic relationship or interaction between 5-HTTLPR and BDNF Val66Met, they also found the S and Met group to be the most vulnerable genetic grouping against a combined non-S and Met group. While an earlier structural MRI study (Pezawas et al. 2008) reported the S and Val combination to be the most vulnerable against other combinations, a more recent study (Carballedo et al. 2012) reported the structural connectivity of the ACC and the AMY to be reduced in Met carriers. Within a multimodal MRI framework, future research should integrate the structural and functional lines of enquiry by examining the impact of the epistasis of 5-HTTLPR and BDNF Val66Met polymorphisms on the relationship between the structure and structural connections of regions involved in emotion processing as well as their function and functional connections.

A main effect for 5-HTTLPR was observed for emotional stimuli such that S carriers had greater activation in the rACC and AMY than L/L homozygotes. Together with the extant literature (see Murphy et al. 2012 for a meta-analysis), S carriers are more reactive to emotional stimuli. While there is debate as to the magnitude of the effect of the 5-HTTLPR polymorphism function on AMY function – Murphy et al. (2012) suggest the effect is smaller in magnitude than previously thought (Munafò et al. 2008) – our data demonstrate a moderate effect of this polymorphism on the AMY and a large effect on the rACC. These findings suggest that the effects of 5-HTTLPR may be stronger in the rACC than in the AMY, which in turn impact on AMY reactivity via reentrant feedback.

We also observed a main effect of BDNF Val66Met in the rACC and AMY, with Met66 carriers showing greater reactivity to emotional stimuli than Val/Val homozygotes; a finding consistent with previous fMRI research on emotion processing (Montag et al. 2008; Mukherjee et al. 2011). Additionally, previous behavioral (Beevers et al. 2009; Terracciano et al. 2010), structural (Pezawas et al. 2004; Carballedo et al. 2012), molecular (Chen et al. 2006), and fMRI memory consolidation (Egan et al. 2003) studies have identified Met66 carriers as being more at risk for affective disorders and related traits. Due to the lower neural plasticity associated with lower BDNF levels and impaired memory consolidation processes, it has been suggested that the BDNF Met66 allele reduces capacity for the retrieval of emotional memories (Mukherjee et al. 2011). This impairment consequently impacts the ability to process the present emotional context, and thus to respond to it adaptively (Mukherjee et al. 2011). The overreactivity displayed in BDNF Met66 allele carriers may be also associated with hyperactivity of neurovisceral networks (including the rACC; Lane et al. 2009) involved in the activation and regulation of the autonomic nervous system (Thayer 2006; Gatt et al. 2009), and our results suggest that these networks may be further and partially moderated by 5-HTTLPR status. Therefore, those carrying the BDNF Met66 allele may have a reduced capacity to strengthen networks that regulate reactivity to emotional stimuli through learning in previous emotional contexts.

Due to the infancy of research in this area (Martinowich and Lu 2008), a limitation that is faced by researchers is small sample size, which is then magnified when attempting to examine epistatic interactions. However, the large effects and clear differences between groups at the individual subject level within a homogenous sample are reassuring. Indeed, significant and strong fMRI findings obtained from smaller samples with conservative correction procedures may be true, rather than false, positives and consistent with findings from larger samples (Murphy and Garavan 2004), particularly within the imaging genetics paradigm (Meyer-Lindenberg et al. 2008). As there are known modulatory effects of hormone status (e.g., Felmingham et al. 2012) and sex on gene–brain (e.g., Everaerd et al. 2012) and gene-affective disorder vulnerability (e.g., Sjöberg et al. 2006), our findings are limited to the female sex only. Future studies employing emotion processing paradigms and recruiting larger, homogenous samples of females and males will broaden and increase confidence in findings on the impact of the functional epistasis of 5-HTTLPR and BDNF Val66Met on brain-behavioral correlates of emotion processing.

In conclusion, our preliminary study demonstrates a role for 5-HTTLPR, BDNF Val66Met, and their epistasis on emotion processing. Building on previous findings, our novel contribution to the literature is an illustration of a potential functional epistasis of 5-HTTLPR and BDNF Val66Met polymorphisms on emotion processing. The functional impact of the BDNF Val66Met allele may be partially dependent on 5-HTTLPR alleles, with the emotional reactivity of the rACC and AMY being implicated in this epistasis. Both Wang et al. (2012) and our findings, in independent samples and tasks, provide support for S and Met as a vulnerable genetic grouping. Future research on the epistasis of 5-HTTLPR and BDNF Val66Met should consider both its structural and functional impacts, employing large, homogenous samples of females and males. Working within a gene–brain–behavior framework, future clinical research should consider the potential for a structural–functional epistasis of 5-HTTLPR and BDNF Val66Met that may underpin vulnerability for affective disorders.
